# Infective Endocarditis in Special Populations: Epidemiology, Diagnostic Challenges, and Management Strategies

**DOI:** 10.7759/cureus.93293

**Published:** 2025-09-26

**Authors:** Abubakar I. Sidik, Maxim A Chinikov, Lyudmila S Korjueva, Eima Abdi, Rostamian Seyedamirali, Alibafghi Mobin, Atahanov Vepa, Kabboudi Hela, Singogo Tumaini, Mdolo Gaudensia, Haya R Abu Sharbeen, Hope Sibalwa, Vivian U Ejim, Masela James, Ali Hassan, Kelala Yasser, Salehk Amro Zuhair Salah, Emmanuel Joachim Njoya Mbombo, Emmanuel S Ndhlovu, Rashed R Ahmad Ameralharthi

**Affiliations:** 1 Cardiovascular Surgery, Peoples' Friendship University of Russia, Moscow, RUS; 2 Department of Hospital Surgery with a Course of Pediatric Surgery, Peoples' Friendship University of Russia, Moscow, RUS; 3 Cardiovascular Medicine, Peoples' Friendship University of Russia, Moscow, RUS; 4 Department of Cardiovascular Medicine, Moscow Multidisciplinary Clinical Center Kommunarka, Moscow, RUS

**Keywords:** cardiac device-related infective endocarditis, congenital heart disease, diagnostic imaging, immunocompromised patients, infective endocarditis, intravenous drug use, surgical management, valve endocarditis

## Abstract

Infective endocarditis (IE) remains a severe cardiovascular infection with persistently high morbidity and mortality despite advances in antimicrobial therapy, imaging, and surgical techniques. Its burden is disproportionately borne by special populations, including patients with prosthetic or transcatheter valves, people who inject drugs, pregnant women, individuals with congenital heart disease, immunocompromised hosts, and critically ill patients. These groups face distinct epidemiological patterns, atypical clinical presentations, and complex management challenges that diverge from native valve disease. We conducted a comprehensive literature review across PubMed/MEDLINE, Web of Science, and Scopus, ultimately including 57 studies addressing epidemiology, diagnostic approaches, surgical management, and outcomes in special populations. Findings highlight increased susceptibility due to prosthetic material, injection practices, immunosuppression, and pregnancy-related physiological changes, as well as diagnostic delays stemming from atypical features or imaging limitations. Management requires balancing infection control with patient-specific risks, including maternal-fetal safety, recurrent infections, or frailty in transplant and ICU patients. Outcomes across groups remain poor, underscoring the need for tailored multidisciplinary strategies. Future directions include refined risk stratification tools, wider adoption of advanced imaging modalities, integration of harm reduction and preventive approaches, and establishment of registries to generate high-quality data in underrepresented populations. By synthesizing current evidence, this review emphasizes the necessity of individualized strategies to improve care and prognosis for vulnerable patients with IE.

## Introduction and background

Infective endocarditis (IE) remains one of the most serious cardiovascular infections worldwide. Despite advances in antimicrobial therapy, surgical techniques, and diagnostic imaging, the disease continues to carry high rates of morbidity and mortality. A 2022 review reports IE prevalence at 5-14.3 per 100,000 adults/year, with in-hospital mortality up to 50% and 19-82% 5-year mortality, driven by aging populations and devices [1-3]. Beyond its direct clinical impact, IE also imposes substantial healthcare costs due to prolonged hospitalizations, repeated surgical interventions, and long-term complications such as heart failure and systemic embolization.

While IE can develop in otherwise healthy individuals, its burden is disproportionately borne by certain “special populations” whose risk profiles, clinical presentations, and outcomes diverge significantly from the general population. These include patients with prosthetic or transcatheter valves, people who inject drugs (PWID), pregnant women, individuals with congenital heart disease, immunocompromised hosts, and critically ill patients such as solid organ transplant recipients or those in intensive care. In these groups, IE is often more aggressive, more difficult to diagnose, and associated with poorer prognoses. Diagnostic uncertainty may stem from atypical presentations or the limitations of conventional imaging in patients with prosthetic material, while therapeutic challenges range from balancing maternal and fetal safety in pregnancy to addressing recurrent infections in PWID or drug-resistant pathogens in immunocompromised patients [2,4,5].

Despite advances in international guidelines, significant gaps remain in the management of IE among special populations. Current recommendations are largely derived from studies in patients with native valve disease, while evidence for groups such as pregnant women, solid organ transplant recipients, immunocompromised patients, and PWID is sparse. As a result, management criteria for these populations often rely on expert opinion or extrapolation from small observational cohorts [2,6,7].

Given these complexities, IE in special populations requires a nuanced and individualized approach that extends beyond the conventional diagnostic and therapeutic frameworks applied to native valve endocarditis. The aim of this narrative review is to synthesize current knowledge on IE in these vulnerable groups, highlighting the distinct epidemiological patterns, diagnostic challenges, and management considerations that shape their care. By drawing attention to these populations, we hope to underscore the need for tailored strategies that can improve outcomes and guide future research.

## Review

Methodology

A comprehensive literature search was conducted using PubMed/MEDLINE, Web of Science, and Scopus. The search strategy incorporated both Medical Subject Headings (MeSH) and free-text keywords to ensure broad coverage of the relevant literature. The primary MeSH term applied was “Endocarditis, Bacterial”. To capture the focus on special populations, additional MeSH terms were included: “Heart Valve Prosthesis”, “Prosthesis-Related Infections”, “Transcatheter Aortic Valve Replacement”, and “Cardiac Devices, Implantable” for prosthetic and device-related endocarditis; “Substance Abuse, Intravenous” and “Drug Users” for PWID; “Pregnancy Complications, Infectious” and “Pregnancy” for pregnancy-associated cases; “Heart Defects, Congenital” and “Adult Congenital Heart Disease” for congenital heart disease; “Immunocompromised Host”, “Organ Transplantation”, “Immunosuppression”, and “HIV Infections” for immunocompromised populations; and “Critical Illness”, “Intensive Care Units”, and “Sepsis” for critically ill patients.

To complement these controlled vocabulary terms, free-text keywords and synonyms were used to identify studies not yet indexed with MeSH. These included IE, prosthetic valve endocarditis, transcatheter valve endocarditis, device-related endocarditis, PWID endocarditis, IVDU endocarditis, maternal endocarditis, congenital heart disease endocarditis, HIV endocarditis, transplant-associated endocarditis, and ICU-associated endocarditis. This combined approach ensured a comprehensive and inclusive capture of the available evidence across multiple special populations.

The initial search retrieved 251 articles. After restricting results to original research articles and clinical guidelines published in English, 195 studies remained. Titles and abstracts were then screened for relevance to the surgical management of IE, and 57 articles were ultimately included for detailed review. Exclusion criteria were conference abstracts, case reports, editorials, narrative reviews, and expert opinion pieces. For each included study, data were extracted from the main text, tables, and figures. In addition, reference lists of the selected articles were manually reviewed to identify further relevant publications. Extracted information was synthesized to provide an overview.

Results

Prosthetic Valve and Transcatheter Valve Infective Endocarditis (IE)

Patients with surgically implanted prosthetic valves and those undergoing transcatheter interventions face a markedly higher risk of IE compared with individuals with native valves. This increased susceptibility reflects both the biological characteristics of prosthetic material and the healthcare exposure associated with implantation procedures [1]. Reported incidence rates of prosthetic valve IE (PVE) range from 0.3% to 1.2% per patient-year, with transcatheter valve endocarditis (TAVI-IE) also emerging as a growing concern as the use of transcatheter aortic valve implantation expands [8,9]. The EURO-ENDO registry reports that prosthetic valve endocarditis (PVE) constitutes 30.1% of IE cases, with an in-hospital mortality rate of 17.1%, higher than the 15.7% for native valve endocarditis [1]. The FinnValve Registry found no difference in PVE risk between transcatheter aortic valve replacement (TAVR) and surgical aortic valve replacement (SAVR) (HR 1.09), with incidences of 3.4 and 2.9 per 1,000 person-years, respectively. However, TAVR-PVE carried a higher in-hospital mortality (60% vs. 32%), potentially due to older, frailer patients [10].

**Figure 1:**
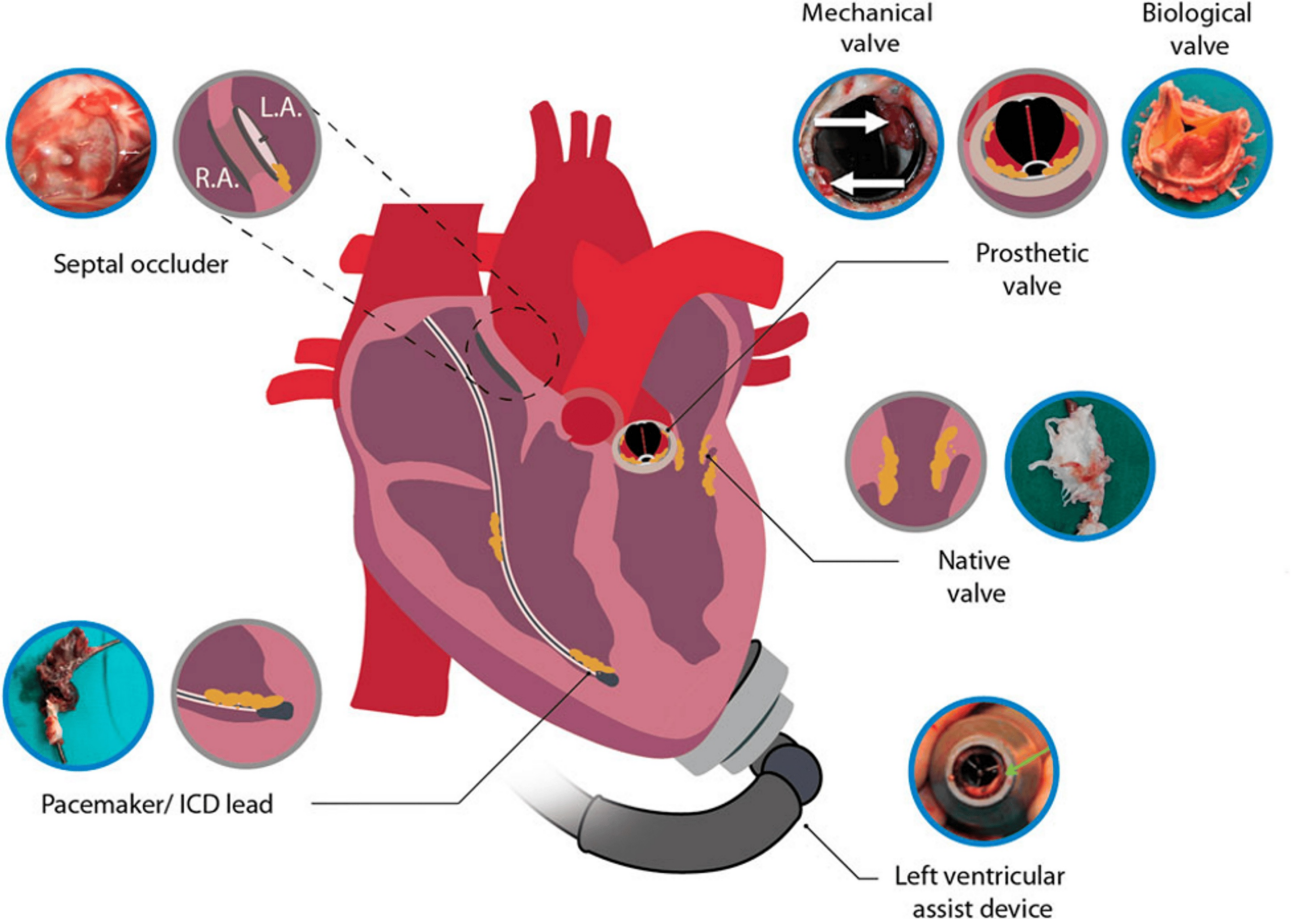
Infective endocarditis associated with native heart tissue and cardiac devices. Included images are examples of an infected septal occlude, mechanical heart valve, biological prosthetic heart valve, native heart valve, LVAD, and pacemaker lead. LA: left atrium; RA: right atrium; LVAD: left ventricular assist device Reprinted under the terms of the Creative Commons Attribution 4.0 International License from Kouijzer et al., Front. Cell Dev. Biol, 2022 [11].

Several mechanisms contribute to this elevated risk. Prosthetic material provides a surface for bacterial adherence and biofilm formation, while procedural contamination during implantation or subsequent interventions may introduce pathogens [6]. Furthermore, the presence of prostheses predisposes to paravalvular extension of infection, leading to abscess formation, dehiscence, and conduction abnormalities that complicate management. Figure 1 illustrates IE manifestations across native valves, prosthetic valves, and cardiac devices, highlighting the diverse sites of infection in special populations [11].

**Figure 2:**
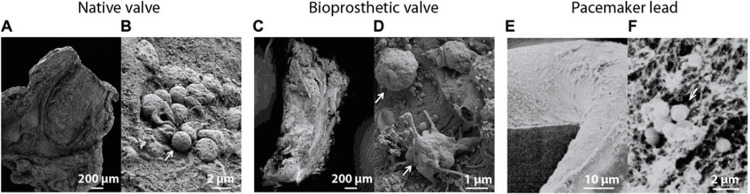
Scanning electron microscopic images of IE A) Infected native heart valve (200 μm) with B) magnified bacteria (2 μm). C) Infected bioprosthetic valve (200 μm) with D) magnified bacteria on fibrous surface (1 μm). E) Biofilm on pacemaker lead (10 μm) with F) magnified bacteria in fibrillar substrate (2 μm). White arrows indicate bacteria. Reprinted under the terms of the Creative Commons Attribution 4.0 International License from Kouijzer et al., Front. Cell Dev. Biol., 2022 [11].

The microbiological profile of PVE and TAVI-IE differs from native valve disease. Staphylococci, particularly Staphylococcus aureus and coagulase-negative staphylococci, are predominant, reflecting the healthcare-associated nature of these infections. Enterococci and non-HACEK Gram-negative organisms also occur more frequently than in native valve cases, underscoring the influence of invasive procedures and prolonged hospital exposure [10,12]. Figure 2 illustrates the anatomical sites of IE, highlighting the complexity of prosthetic and device-related infections [11].

Clinical manifestations are often subtle, and diagnosis may be delayed. Standard echocardiography can be limited by shadowing from prosthetic material, reducing sensitivity in detecting vegetations or paravalvular complications. Advanced imaging techniques, including positron emission tomography/computed tomography (PET/CT) and cardiac CT, have improved diagnostic accuracy but remain underutilized in routine practice [13-15].

Outcomes in this population are notably worse. PVE and TAVI-IE are associated with higher in-hospital mortality, frequent complications such as heart failure and systemic embolization, and a greater need for surgical intervention. Even after surgery, the risk of recurrence remains elevated, reflecting both patient frailty and the challenges of eradicating biofilm-associated infection [16,17].

Several strategies hold promise in improving outcomes in prosthetic valve IE, where surgical needs are higher, early intervention (within 20 days) significantly lowers mortality (OR 0.57 for all-cause, 95% CI 0.34-0.96), supporting aggressive evaluation despite patient frailty [8]. Enhanced imaging protocols may facilitate earlier and more accurate diagnosis, while refinements in prosthetic and transcatheter valve design could reduce susceptibility to microbial colonization [18]. Preventive approaches, including strict peri-procedural aseptic measures, targeted antimicrobial prophylaxis, and long-term patient education, remain central to risk reduction [1]. Ongoing research into biomaterials with antimicrobial properties and minimally invasive re-intervention techniques is shifting the balance in favor of better outcomes for this high-risk group.

Infective Endocarditis in People Who Inject Drugs

The incidence of IE among PWID has risen sharply in many high-income countries over the past two decades, paralleling the opioid epidemic. In some regions, PWID now account for up to one-third of new IE cases. This epidemiological shift has profound implications for healthcare systems, as these patients are typically younger, have fewer comorbidities, but present with highly complex disease courses and high recurrence rates [19,20].

The pathophysiology reflects the unique exposure risks associated with injection practices. Repeated vascular access allows direct inoculation of microorganisms, most often skin flora such as Staphylococcus aureus. The right heart is disproportionately affected, with the tricuspid valve being the predominant site of infection. The hemodynamic and anatomical features of the right heart make it particularly susceptible to septic pulmonary emboli, which frequently complicate the clinical course [21,22].

Clinically, PWID-IE often manifests with fever, persistent bacteremia, and respiratory symptoms related to septic embolization. While left-sided involvement is less common, when present, it carries significantly higher morbidity and mortality. Recurrence is a major challenge, as reinfection rates approach 30% in some cohorts, driven by ongoing injection behavior and repeated exposure to healthcare environments [23,24].

Surgical management in this group remains controversial. Tricuspid valve repair is generally preferred when feasible, as it avoids prosthetic material and preserves right ventricular function. When replacement is necessary, bioprosthetic valves are favored over mechanical options to avoid the need for long-term anticoagulation, which is poorly tolerated in patients at risk of recurrent infection or poor adherence [25]. Valvectomy without replacement may be considered in select critically ill patients but carries long-term risks of right heart failure. The balance between immediate survival and long-term outcomes often shapes surgical decision-making [26,27].

Beyond antimicrobial therapy, timely surgical intervention is critical; a 2016 meta-analysis of 21 studies found early surgery (within 20 days) reduced all-cause mortality by 39% compared to delayed or medical approaches (OR 0.61, 95% CI 0.50-0.74) [8]. Optimal care extends beyond infection control and surgery. A multidisciplinary approach involving cardiology, cardiac surgery, infectious diseases, addiction medicine, and social services is essential [22,28,29]. Harm reduction strategies, including needle exchange programs, opioid substitution therapy, and structured rehabilitation, play a pivotal role in breaking the cycle of reinfection [30,31].

Pregnancy-Associated IE

IE during pregnancy is uncommon, but when it occurs, the consequences are often catastrophic. Reported maternal mortality approaches 18%, while fetal loss has been estimated at nearly 29%, underscoring the dual risks to both mother and child. The rarity of the condition, coupled with its severity, makes it one of the most challenging scenarios in contemporary cardiovascular and obstetric practice [32-34].

Predisposing factors: Several predisposing factors converge to increase susceptibility in this population. Women with congenital heart disease (CHD), prosthetic valves, or a history of rheumatic heart disease remain particularly vulnerable, especially in regions where rheumatic disease persists as a significant health burden. In high-income countries, PWID represent an additional subgroup at heightened risk. The rising survival of women with complex CHD into adulthood has also contributed to an increasing pool of pregnant patients predisposed to IE [35,36].

Diagnostic challenges: Diagnosis is often delayed because many of the cardinal symptoms of IE, fatigue, dyspnea, and palpitations, overlap with normal physiological changes of pregnancy. Fever, anemia, and murmurs may be mistakenly attributed to pregnancy-related conditions, leading to under-recognition. Imaging adds another layer of complexity: transthoracic echocardiography is safe but less sensitive in detecting vegetations, while transesophageal echocardiography and nuclear imaging carry concerns about fetal exposure, limiting their use [37,38].

Management dilemmas: Management requires balancing maternal and fetal risks at every stage. Antibiotic selection must take into account teratogenic potential and altered pharmacokinetics during pregnancy [39]. When surgical intervention becomes unavoidable, cardiopulmonary bypass poses serious hazards, with maternal mortality estimated at 7-15% and fetal mortality exceeding 25%. Timing of delivery relative to surgical intervention adds further complexity, often requiring individualized decision-making based on gestational age, maternal stability, and infection severity [40,41].

Importance of multidisciplinary “Endocarditis & Obstetric Team.” The need for a dedicated multidisciplinary approach cannot be overstated. An integrated “Endocarditis and Obstetric Team”-including cardiologists, cardiac surgeons, obstetricians, anesthesiologists, infectious disease specialists, and neonatologists-optimizes both maternal and fetal outcomes. Such teams can coordinate timely imaging, tailor antibiotic regimens, and plan surgical or obstetric interventions with a shared understanding of risks [42,43].

Preventive strategies: Prevention represents a critical frontier. Preconception counseling for women with CHD or prosthetic valves can guide risk stratification and contraception choices. Screening high-risk women early in pregnancy, ensuring adherence to antibiotic prophylaxis when indicated, and providing education on infection prevention are essential steps. Strengthening collaboration between cardiology and maternal health services offers the best opportunity to reduce the devastating toll of pregnancy-associated IE [44,45].

Congenital heart disease (CHD) and IE

The population of adults living with CHD has grown substantially in recent decades, reflecting advances in pediatric cardiac surgery and long-term medical care. This demographic shift has been accompanied by a rising burden of IE, which is now recognized as a significant late complication in this group. Adults with CHD face an incidence of IE many times higher than that of the general population, and the condition often presents with greater complexity due to underlying anatomical and surgical factors [46,47].

Risk factors: Several risk factors contribute to this heightened susceptibility. The presence of prosthetic material, such as conduits, shunts, or prosthetic valves, creates surfaces prone to bacterial adherence and biofilm formation. Residual lesions after surgical or percutaneous repair, persistent cyanosis, and the presence of multiple structural defects further amplify risk. In particular, patients with complex lesions requiring multiple staged interventions remain vulnerable throughout their lifespan [48,49].

Right-sided predominance and delayed diagnosis: IE in CHD frequently demonstrates a right-sided predominance, especially in those with palliative shunts or repaired lesions involving the right ventricular outflow tract. Diagnosis is often delayed, partly because clinical manifestations may be attributed to baseline cardiac abnormalities, and because imaging is technically challenging in patients with distorted anatomy or prosthetic material. As a result, complications such as abscesses, embolic events, or worsening heart failure may already be present at the time of diagnosis [50,51].

Variability in risk between lesions: Importantly, not all CHD lesions carry the same risk of IE. Patients with bicuspid aortic valve disease have a lower, though still elevated, risk compared with the general population, whereas those with repaired ventricular septal defects may have risk confined largely to the first six months after closure, unless residual defects persist. In contrast, cyanotic CHD, prosthetic right-sided conduits, and complex multilevel lesions are associated with a lifelong and substantial risk [52].

Long-term considerations: Long-term considerations are central to managing IE in this population. Repeated surgical and catheter-based interventions not only increase exposure to healthcare-associated pathogens but also add more prosthetic material, perpetuating the cycle of risk [53]. The role of antibiotic prophylaxis remains an area of debate, particularly as guidelines have narrowed recommendations to specific high-risk groups. Balancing the risks of prophylaxis against the devastating consequences of IE requires individualized assessment, informed by lesion type, prior interventions, and patient-specific vulnerabilities [54,55].

IE in Immunocompromised Patients

Immunocompromised populations represent a particularly vulnerable group for IE, with atypical clinical features, challenging diagnoses, and poorer outcomes compared with immunocompetent patients. The spectrum of risk includes solid organ transplant recipients, individuals living with HIV, and patients with other forms of iatrogenic or disease-related immunosuppression [56-58].

Solid Organ Transplant Recipients

The incidence of IE in solid organ transplant recipients is estimated at 1-2%, reflecting both prolonged immunosuppression and frequent exposure to healthcare interventions. Unlike classic valve-related disease, these patients often develop mural endocarditis without direct valve involvement, which can delay recognition and complicate management. Staphylococcus aureus is the predominant pathogen, consistent with its invasive and healthcare-associated nature [59].

Prognosis is especially poor when IE occurs during the index transplant hospitalization, a period characterized by heightened immunosuppression, surgical stress, and prolonged device use. Surgical referral rates remain low due to concerns over frailty, graft function, and postoperative risks [60]. Mortality is further amplified in cases of fungal IE, which are notoriously resistant to therapy and difficult to eradicate. Overall, outcomes highlight the need for vigilance, early imaging, and aggressive infection control in this population [61].

HIV and Other Immunosuppressed States

The epidemiology of HIV-associated IE has shifted in the era of effective antiretroviral therapy, with declining incidence compared to earlier decades. Nonetheless, patients with HIV remain vulnerable, particularly those with advanced disease or poor adherence to therapy. The coexistence of multiple comorbidities, such as viral hepatitis and substance use, adds further complexity. Healthcare-associated IE is also more frequent in this population, reflecting recurrent hospitalizations and indwelling devices [58].

In other immunosuppressed states, including chemotherapy-induced neutropenia, hematologic malignancies, and long-term corticosteroid therapy, the microbiological spectrum broadens. Viridans group *Streptococci *remain common, but fungal pathogens such as *Candida *and *Aspergillus *are disproportionately represented in neutropenic patients, often with devastating outcomes. These infections tend to present atypically, lack classic vegetation on imaging, and carry very high mortality despite aggressive therapy [56,57]. Table 1 details pathogen prevalence, showing *Staphylococci *dominance in PVE (32%) and fungi’s 72% mortality in cardiac device-related IE (CDRIE) [11].

**Table 1:** Microbiological causes of native valve (NVE), prosthetic valve (PVE), and cardiac device-related infective endocarditis (CDRIE), with prevalence and mortality rates. Reprinted under the terms of the Creative Commons Attribution 4.0 International License from Kouijzer et al., Front. Cell Dev. Biol., 2022 [11]. HACEK: *Haemophilus*, *Aggregatibacter*, *Cardiobacterium*, *Eikenella*, and *Kingella*

Pathogen	Prevalence in NVE (%)	Prevalence in PVE (%)	Prevalence in CDRIE (%)	Mortality Rate (%)	Notes
Staphylococci (e.g., *S. aureus*)	27	32	35	~20–30	Predominant in PVE/CDRIE; biofilm-forming.
Streptococci	30	25	15	~10–15	Common in NVE; oral cavity origin.
Enterococci	10	16	12	~15–20	Increasing in PVE; nosocomial link.
Fungi (e.g., *Candida*, *Aspergillus*)	2	5	8	72	Rare but deadly; common in immunocompromised.
Culture-Negative	10	15	20	~20–25	Challenges with atypical pathogens.
Other (e.g., HACEK, Gram-negative)	21	7	10	~15–20	Diverse; context-specific.

**Figure 3:**
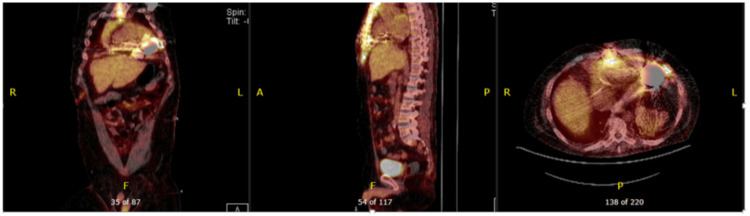
LVAD (HeartMate 3) with Candida albicans infection. 18F-FDG PET-CT showed FDG activity in the pericardium, mediastinum, surrounding LVAD pump, driveline, and sternum consistent with deep and superficial LVAD infection. Reprinted under the terms of the Creative Commons Attribution 4.0 International License from Aguilera et al., Front. Cardiovasc. Med., 2021 [62]. LVAD: left ventricular assist device

Figure 3 illustrates 18F-FDG PET-CT’s ability to detect extensive left ventricular assist device (LVAD) infection, guiding therapy in critically ill patients [62].

Critically Ill Patients and ICU-Associated IE

High overlap with healthcare-associated infections: In the ICU, IE often emerges in the context of healthcare-associated infections, reflecting the high prevalence of invasive procedures, indwelling devices, and exposure to broad-spectrum antimicrobials. Critically ill patients are uniquely predisposed to bloodstream infections, and distinguishing IE from other sources of sepsis remains a major clinical challenge [63,64].

The challenges were differentiating from other sepsis sources, late diagnosis: diagnosis is frequently delayed because fever, leukocytosis, and hemodynamic instability are nonspecific findings in this population. Many patients have multiple potential infection sources, including ventilator-associated pneumonia, central line infections, and urinary tract infections, making it difficult to attribute persistent bacteremia to endocardial involvement. This delay contributes to the progression of disease and poorer outcomes [65,66]. Diagnostic challenges in prosthetic valve IE include limited echocardiography sensitivity due to metal artifacts. Table 2 compares modalities, showing [18F] FDG PET/CT’s 91% sensitivity for PVE, surpassing transthoracic echocardiography (TTE) (63%) and TEE (84%) [11]. This aligns with the 2023 Duke-ISCVID criteria, advocating PET/CT for complex cases, though availability remains a barrier [10].

**Table 2:** Diagnostic accuracy with reported sensitivity and specificity of various imaging modalities. ᵃOnly reported once since 2016. CDRIE: cardiac device-related infective endocarditis; CT: computed tomography; [¹⁸F] FDG: 18F-fluorodeoxyglucose; IE: infective endocarditis; NVE: native valve endocarditis; PET: positron emission tomography; PVE: prosthetic valve endocarditis; Se: sensitivity; Sp: specificity; SPECT: single photon-emission computed tomography; TEE: transesophageal echocardiography; TTE: transthoracic echocardiography; WBC: white blood cell Reprinted under the terms of the Creative Commons Attribution 4.0 International License from Kouijzer et al., Front. Cell Dev. Biol, 2022 [11].

	All IE		NVE		CDRIE		PVE	
Imaging	Se (%)	Sp (%)	Se (%)	Sp (%)	Se (%)	Sp (%)	Se (%)	Sp (%)
TTE	71 (60–82)	57ᵃ	84 (70–98)	93ᵃ	17ᵃ	100ᵃ	63 (60–65)	79 (67–95)
TEE	81 (36–95)	70 (42–85)	94 (91–98)	78 (67–88)	67ᵃ	100ᵃ	84 (78–91)	67 (57–75)
[18F]FDG PET/CT	81 (74–88)	86 (79–92)	36 (17–68)	97 (85–100)	82 (56–96)	90 (80–100)	91 (75–100)	67 (29–93)
WBC SPECT/CT	90 (86–100)	98 (95–100)	—	—	73 (60–84)	87 (74–100)	—	—
CT	53 (16–89)	84 (71–96)	49 (11–80)	77 (63–92)	75ᵃ	86ᵃ	73 (19–96)	78 (50–98)

Frequent device-related IE: Device-related infections are particularly common. Central venous catheters, long-term vascular access, and intracardiac devices such as left ventricular assist devices and prosthetic materials are frequent entry points for pathogens. Staphylococci, including Staphylococcus aureus and coagulase-negative species, dominate the microbiological landscape, consistent with device-associated and healthcare-acquired infections [5,67].

Mortality: Despite improvements in critical care and infection control, outcomes for ICU-associated IE remain poor. Mortality rates are high, often exceeding those observed in community-acquired IE, reflecting both the severity of underlying illness and the complexity of managing infection in unstable patients. Surgical options are often limited due to frailty, multi-organ dysfunction, or poor operative candidacy [65,66].

**Figure 4:**
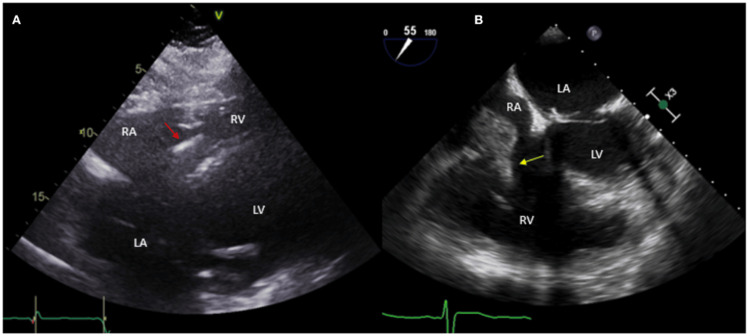
ICD patient presented with fever and methicillin-sensitive Staphylococcus aureus (MSSE) bacteremia. Initial TTE done. A) subcostal view with ICD lead in place (red arrow), without evident vegetations. Based on high clinical suspicion for cardiac implantable electronic device infection, a TEE was obtained, which showed a large vegetation attached to the ICD lead prolapsing into the right ventricle (yellow arrow). ICD: Implantable cardioverter-defibrillator Reprinted under the terms of the Creative Commons Attribution 4.0 International License from Aguilera et al., Front. Cardiovasc. Med., 2021 [62].

Strategies: Addressing these challenges requires systematic strategies. Early use of echocardiography, particularly transesophageal studies, should be incorporated into sepsis protocols when bacteremia persists or when device-related infection is suspected. The diagnostic superiority of TEE over TTE in device-related IE is evident in Figure 4, where vegetation was missed on TTE but confirmed on TEE, leading to extraction [62]. Advanced imaging, including CT and PET/CT, may help identify subtle complications. Standardized sepsis pathways that include consideration of IE can facilitate earlier recognition and treatment. Preventive measures, such as meticulous device care, timely catheter removal, and strict adherence to aseptic protocols, remain cornerstones of reducing risk [63,65].

Future Directions

Managing IE in special populations requires moving beyond conventional frameworks and adopting strategies tailored to their unique risks. Several key avenues are emerging that may reshape prevention, diagnosis, and treatment in the coming years [6,68].

Better risk stratification for special populations: Better risk stratification remains a priority. Current clinical tools inadequately capture the complexity of patients with prosthetic valves, CHD, immunosuppression, or substance use disorders. Developing refined models that incorporate demographic, microbiological, and procedural variables could help identify those at greatest risk and guide targeted surveillance or prophylaxis [69].

**Figure 5:**
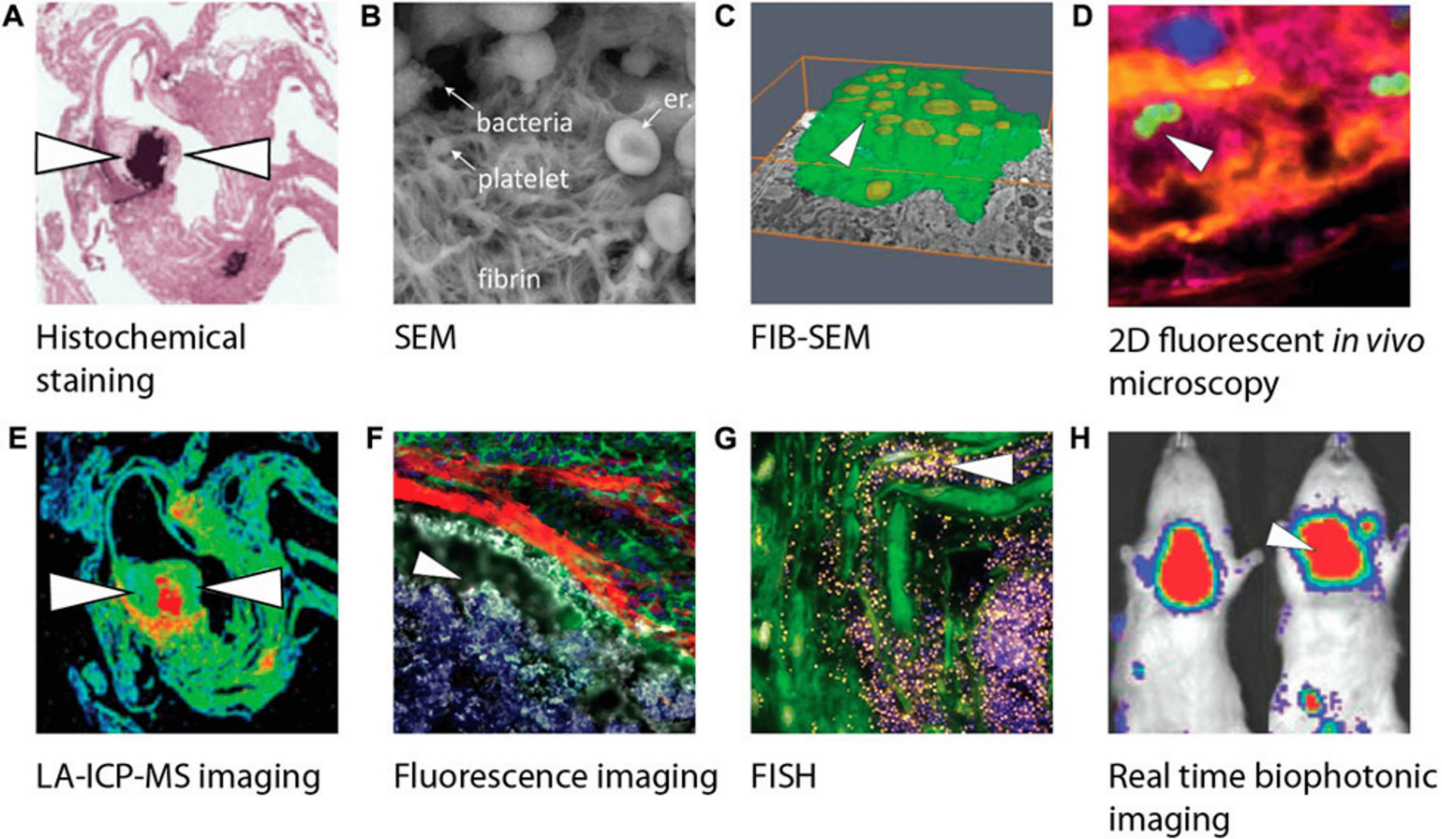
Overview of biofilm visualization modalities in IE research A) Crystal violet staining of bacteria in mouse heart tissue. B) SEM image of bacteria in a platelet-fibrin network. C) 3D FIB-SEM of infected phagocytic cells. D) Confocal image of early IE lesions with *S. aureus*. E) LA-ICP-MS elemental map of infected tissue. F) Fluorescence imaging of myeloid cells blocked by fibrin. G) FISH visualization of *S. epidermidis* on valve tissue. H) Bioluminescent imaging of *S. aureus* in rats. White arrowheads indicate bacteria. Images adapted with permission from original publishers. SEM: scanning electron microscopy; FIB-SEM: Focused ion beam SEM; LA-ICP-MS: Laser ablation inductively coupled plasma mass spectrometry; FISH: Fluorescence in situ hybridization Reprinted under Creative Commons Attribution 4.0 International License from Kouijzer et al., Front. Cell Dev. Biol., 2022 [11].

Advances in imaging: Advances in imaging are also transforming the diagnostic landscape. Techniques such as PET/CT and three-dimensional echocardiography offer improved sensitivity for detecting prosthetic valve infection, paravalvular extension, and subtle vegetations that may be missed with standard modalities. Wider adoption of these tools, supported by structured protocols, could shorten diagnostic delays and improve clinical decision-making [13,15]. Emerging research techniques for biofilm visualization, as shown in Figure 5, offer insights into IE pathogenesis and could inform novel diagnostic pipelines [11].

Preventive approaches: Preventive approaches remain central. Strengthening infection control practices in hospitals and ICUs, ensuring meticulous device care, and implementing timely removal of unnecessary catheters are critical. Expanding access to addiction services, including harm reduction programs and medication-assisted treatment, may significantly reduce recurrence rates among PWID. Parallel efforts in biomedical engineering, such as the development of prosthetic materials resistant to biofilm formation, may help reduce susceptibility in valve recipients [4,69].

Need for registries and multicenter studies focusing on underrepresented groups: Finally, there is an urgent need for high-quality data focused on these underrepresented groups. Most randomized trials in IE exclude pregnant women, transplant recipients, and PWID, leaving clinicians reliant on observational data and expert opinion. National and international registries, as well as multicenter collaborative studies, are essential to generate robust evidence that can inform practice and guideline development [4,19].

Limitations

This review has several limitations. First, the included studies were heterogeneous in design, patient populations, and outcome measures, limiting direct comparisons across special populations. Most available evidence is derived from observational studies, registries, or retrospective cohorts, with few randomized controlled trials, especially in underrepresented groups such as pregnant women, solid organ transplant recipients, and critically ill patients. Selection bias and publication bias may also have influenced the literature, as severe or unusual cases are more likely to be reported, while negative or inconclusive findings may remain unpublished.

Diagnostic and therapeutic approaches varied across regions and time periods, reflecting differences in healthcare infrastructure, imaging availability, and local guidelines, which may restrict the generalizability of findings. Finally, despite an extensive search strategy, some relevant studies may not have been captured, particularly those published in languages other than English or outside indexed databases. These limitations underscore the need for high-quality multicenter prospective studies and registries to strengthen the evidence base for managing IE in special populations.

## Conclusions

IE in special populations poses unique challenges that extend beyond the frameworks established for native valve disease. Patients with prosthetic or transcatheter valves, PWID, pregnant women, those with CHD, immunocompromised individuals, and critically ill patients face distinct epidemiological risks, diagnostic barriers, and therapeutic dilemmas. Despite advances in imaging, surgery, and antimicrobial therapy, outcomes in these groups remain disproportionately poor. Addressing this unmet need requires individualized management strategies, multidisciplinary collaboration, and integration of preventive measures such as harm reduction programs, infection control, and preconception counseling in at-risk women. Future progress will depend on improved risk stratification tools, wider adoption of advanced diagnostic modalities, innovation in biomaterials, and the establishment of prospective registries and multicenter studies to generate stronger evidence. By tailoring care to the unique vulnerabilities of these populations, clinicians can improve outcomes and reduce the global burden of this devastating disease.
